# Fabrication and characterization of walnut protein-polyphenol conjugates from hickory by-products: Structure and interaction mechanism analysis

**DOI:** 10.1016/j.fochx.2026.104491

**Published:** 2026-09-21

**Authors:** Shuaizheng Wang, David Julian McClements, Hao Hu, Li Huang, Liang Cao, Changling Wu

**Affiliations:** a Department of Food Science, Zhejiang Agriculture and Forestry University, Hangzhou, Zhejiang 311300, China; b Ningbo Tianyun Food Co., Ltd, Ningbo, Zhejiang, 315700, China; c National Grain Industry (High-Quality Rice Storage in Temperate and Humid Region) Technology Innovation Center, Hangzhou 311300, China; d Department of Food Science, University of Massachusetts, Amherst, MA 01003, USA; e Department of Food Science, Northeast Agricultural University, Harbin, Heilongjiang 150030, China

**Keywords:** Protein-polyphenol interaction, Walnut protein, Free radical grafting, Hickory by-products, Structural modification

## Abstract

The protein-polyphenol interaction mechanisms are affected by component chemical structures, environmental conditions, and processing methods. In this study, walnut protein (WP)-polyphenol complexes or conjugates were prepared using five approaches: physical mixing, heat treatment, alkali treatment, free radical grafting (FRG), and ultrasound-assisted free-radical grafting (UFRG). All samples had amorphous characteristics and FRG/UFRG groups showed a distinct ester bond absorption peak (FTIR: 1739 cm^−1^). Atomic force microscopy showed differences in the aggregation states of complexes and conjugates, with the UFRG group having the smallest aggregates. Raman spectroscopy indicated that under the UFRG treatment, the WP-HSC sample had the highest random coil content (74.30%), highest tryptophan residue exposure (I₇₆₀/I₁₀₀₃ = 1.10), and a near equilibrium conformation of disulfide bonds (g-g-t/t-g-t). Correlation analysis showed the random coil content of WP was significantly positively correlated (*P* < 0.05) with grafting efficiency, turbidity, contact angle and surface hydrophobicity. In summary, this study provides valuable insights into factors regulating plant protein-polyphenol conjugate formation.

## Highlights

• The interaction between polyphenols from hickory by-products and walnut protein was explored.
• Elucidated binding modes of protein-polyphenol in amorphous products.
• UFRG optimized structure by enhancing random coil content and Trp exposure.
• Random coil content correlated positively with grafting rate and hydrophobicity.

## Graphical abstract

**Figure:**
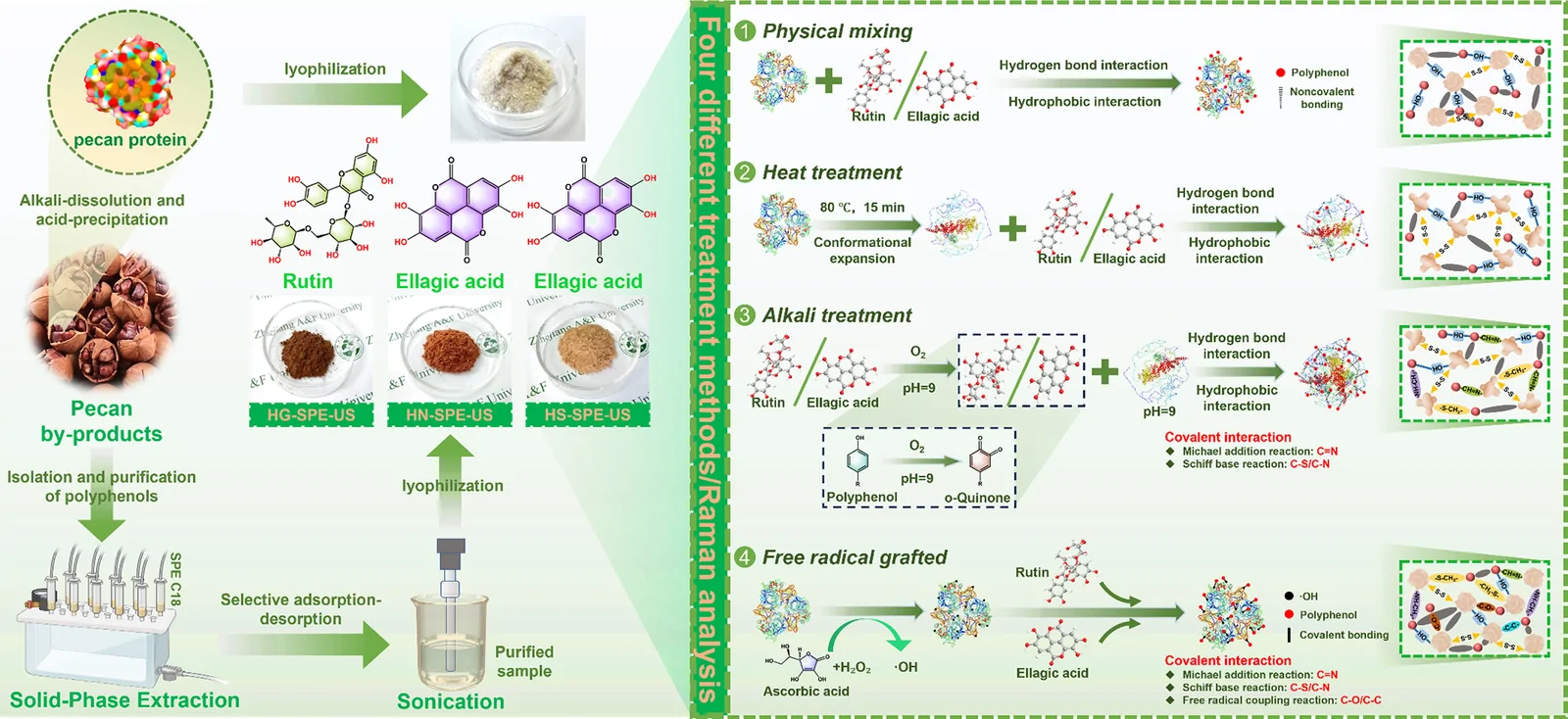

## 1 Introduction

Plant polyphenols are a class of secondary metabolites that are widely distributed in the plant kingdom, which have been reported to exhibit a range of potentially beneficial bioactivities, including antioxidant, anti-inflammatory and anti-tumor properties, as well as the ability to regulate lipid metabolism and improve glucose homeostasis (Hashemi et al., 2025; Huang et al., 2024; Wu et al., 2024). They can also interact with biological macromolecules such as proteins and nucleic acids, scavenge reactive oxygen species (ROS), and regulate the gut microbiota (Loo et al., 2020; Ye et al., 2025). Therefore, these plant polyphenols have emerged as health-promoting ingredients in functional foods and beverages. However, many isolated phenolic substances have bitter tastes, low solubilities, and poor stabilities, which restricts their direct application in food formulations (Soares et al., 2018). Therefore, strategies of protein-polyphenol complexation and covalent conjugation are being increasingly explored for their potential to overcome these drawbacks. Previous studies have shown that food-grade proteins may be good carriers for polyphenols (Ma et al., 2025).

Hickory (*Carya cathayensis Sarg.*), a perennial deciduous fruit tree of the Juglandaceae family, is highly valued for its nuts, which are rich in nutrients and have several potential health benefits (Wu et al., 2024; Wu et al., 2025). However, the processing of hickory generates substantial amounts of inedible byproducts, including hickory husk (HH), hickory shell (HS), or hickory seed coat (HSC). These byproducts are commonly utilized as animal feed or disposed of as waste, raising concerns regarding sustainability and economic efficiency. In our previous research, phenolic compounds were extracted and purified from these three different fractions using solid-phase extraction coupled with ultrasonication. They were then characterized using high-performance liquid chromatography (HPLC) to identify the principal phenolic constituents in the different polyphenol-rich extracts: rutin (HH), ellagic acid and sinapic acid (HS), as well as ellagic acid and ferulic acid (HSC) (Wu et al., 2024). Rutin has a pentahydroxyflavone core linked to a rutinose side-chain *via* a glycosidic bond (Rashidinejad et al., 2019). The molecular structure of ellagic acid exhibits the typical characteristics of hydrolysable tannins with a bicyclic lactone-polyphenolic hydroxyl group (Huang et al., 2024). Sinapic acid and ferulic acid have a C6-C3 hydroxycinnamic acid as their basic skeleton, which consists of a benzene ring and an acrylic acid side chain. The benzene rings are modified with methoxy groups (-OCH₃), phenolic hydroxyl groups (-OH), and terminal carboxyl groups (-COOH) (Fig. 1A∼D) (Hashemi et al., 2025; Yang et al., 2021). These polyhydroxy structures can effectively scavenge free radicals through proton transfer in a polar environment and provide binding sites for the formation of hydrogen bonds with molecules containing functional groups (N-H/O-H). In addition, carboxyl groups (-COOH) can covalently bind to proteins through reactions such as amidation and esterification (Wang, Tan, et al., 2023; Wu et al., 2025). However, like many plant polyphenols, these hickory-derived phenolic compounds also exhibit poor solubility and stability, limiting their direct application in foods. Therefore, a suitable protein carrier is needed to overcome these drawbacks.

**Fig. 1:**
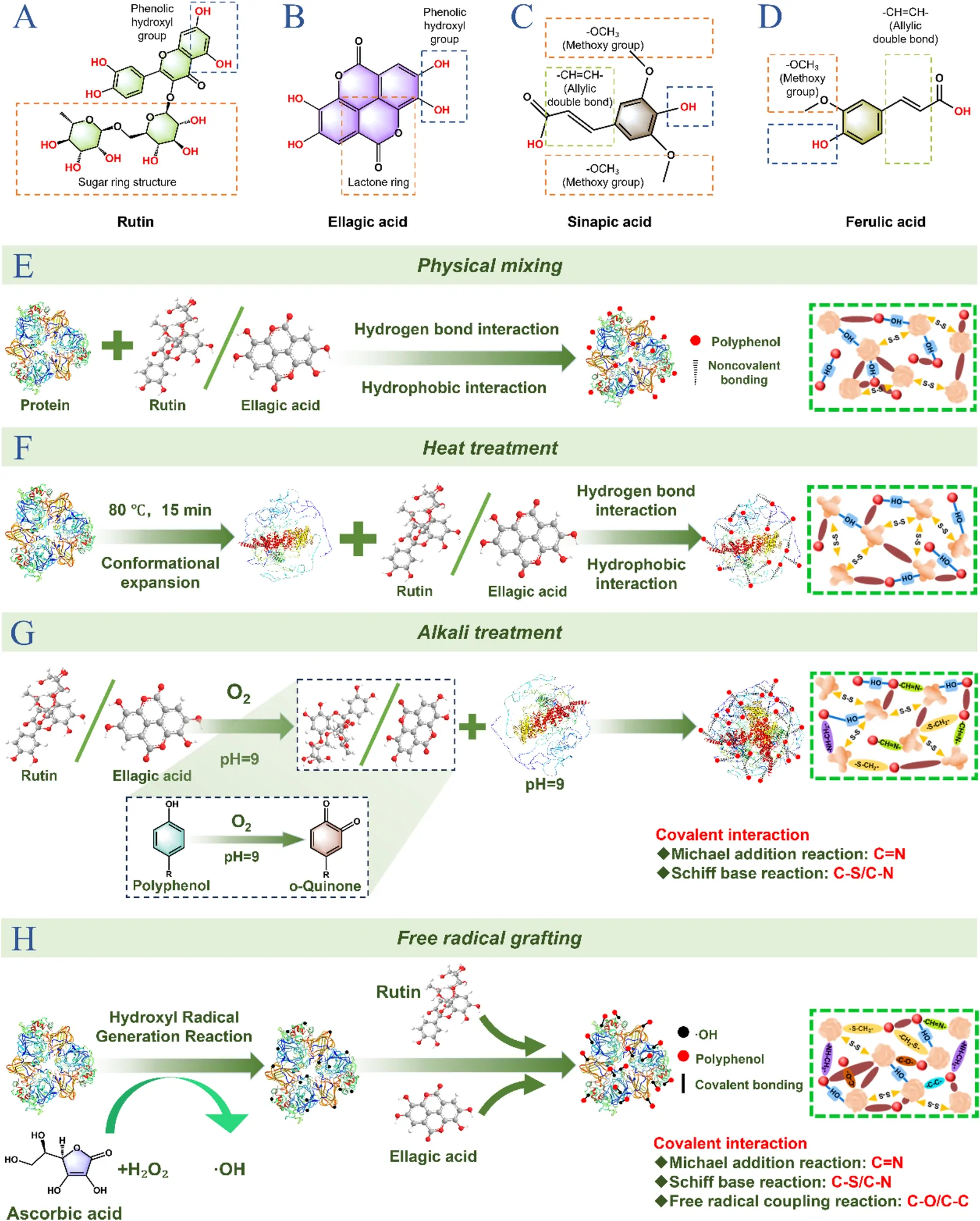
Chemical structural formulas of the main phenolic substances in hickory by-products and mechanism of interaction in protein-polyphenol complexes. (A) Rutin (Rt); (B) Ellagic acid (EA); (C) Sinapic acid (SA); (D) Ferulic acid (FA); (E ∼ H) represent the schematic diagram of interactions for protein-polyphenol complexes in the four treatment groups of physical mixing (PM), heat treatment (HT), alkali treatment (AT), and free radical grafting (FRG), respectively.

Walnut protein (WP) has emerged as an important source of plant proteins due to its balanced essential amino acid composition and relatively low raw material costs (Zhang et al., 2024). WP is mainly composed of glutelin (around 72%) and globulin (around 16%) (Zhang et al., 2024; Zhao et al., 2023). Under neutral pH conditions, WP exists as a compact globular protein held together by non-covalent interactions (Ma et al., 2024). It has an internal hydrophobic cavity, as well as several hydrophobic patches on its surface, which act as binding sites for hydrophobic ligands, including polyphenols (Wang, Chen, et al., 2023). Therefore, WP can be used for the encapsulating, carrying, and delivering hydrophobic bioactives, which is one useful for the development of low-allergenic functional foods (Ma et al., 2024).

In our previous study, we successfully prepared oil-in-water nanoemulsions stabilized by walnut protein-polyphenol conjugates (Wu et al., 2025). These nanoemulsions had high encapsulation efficiencies and good storage stabilities, which was mainly attributed to the presence of the protein-polyphenol conjugates at the oil droplet surfaces. However, the polyphenols from hickory by-products are complex mixtures composed of a diverse range of substances with different chemical structures and contents. We therefore hypothesized that the interactions of the polyphenol-rich extracts with walnut proteins, as well as their impact on the functional properties of protein-polyphenol conjugates, would depend on their origin. Previous studies have mainly focused on the interactions between pure proteins and pure phenolic compounds, while research on the interactions between proteins and complex polyphenol-rich plant extracts is still relatively scarce. Accordingly, five identical treatment protocols from our previous studies were adopted to complex WPs with polyphenol fractions of various hickory by-products for building protein-polyphenol systems comprising non-covalent complexes or covalent conjugates, which permitted deciphering the molecular interaction mechanism behind protein-polyphenol reconstruction and filling gaps in current research. Spectroscopy, light scattering, microscopy, and surface hydrophobicity analyses were then performed to characterize the nature of the complexes and conjugates formed. Correlation analysis was utilized to clarify the core influencing factors that regulate the structure and function of the prepared conjugates or complexes.

## 2 Materials and methods

### 2.1 Materials

Hickory (*Carya cathayensis Sarg.*) was purchased in 2025 from the commercial hickory production base in Linan, Zhejiang, China. These hickory kernels were then defatted using a hydraulic oil press (6YY-460, Zhengzhou Penguin Grain and Oil Machinery Co., Ltd., Zhengzhou, China). The by-products from different parts of hickory (hickory husk, HH; hickory shell, HS; hickory seed coat, HSC) were provided by Hangzhou Opal Technology Co., Ltd. (Shangcheng District, Hangzhou, China). Hydrogen peroxide (H_2_O_2_, CAS: 7722-84-1, 5 M) and ascorbic acid (C₆H₈O₆, CAS: 50–81-7, 98% purity) were purchased from Hebei Chuangzhiyuan Biotechnology Co., Ltd. (Shi Jiazhuang, China). All other chemical reagents used in this experiment are of analytical-grade purity unless otherwise specified.

### 2.2 Preparation of walnut proteins (WPs)

Walnut kernels with their seed coats removed were degreased using a hydraulic oil press to obtain defatted walnut protein flour, following the method described by Ma et al. (2024), with minor modifications. Briefly, the walnut flour was mixed with petroleum ether (boiling range: 30–60 °C) at a 1:10 (*w*/*v*) ratio and then stirred for 3 h to ensure complete defatting. This procedure was repeated until the filtrate became clear. The obtained defatted walnut flour was then stored in a vacuum desiccator for subsequent use. Defatted walnut flour was then mixed with deionized (DI) water at a 1:20 (*w*/*v*) ratio and the pH was adjusted to 11.0 using 1.0 M NaOH. The mixture was then stirred continuously at 55 °C for 1 h, followed by centrifugation at 4000 ×*g* for 20 min at 4 °C. The supernatant was then collected, and its pH was adjusted to 4.5 using 1.0 M HCl. The acidified solution was then centrifuged again at 6000 ×*g* for 20 min at 4 °C. The obtained precipitate was redissolved in a small volume of DI water, and the pH was readjusted to 7.0 using 1.0 M NaOH. Finally, the protein solution was lyophilized (FD-lab-10, Beijing Songyuan Huaxing Technology, Beijing, China) to obtain a light-yellow WP powder. The crude protein content of WP (*N* × 6.25) was determined to be 92.43% using the Kjeldahl method.

### 2.3 Preparation of hickory phenolic compounds

The target phenolic compounds were isolated from HH, HS, and HSC using a C18 solid-phase extraction column (SPE) for purification, combined with the continuous-mode ultrasonic-assisted extraction (300 W, 10 min, 6 mm probe diameter, 50 mL sample volume) technique, following the method established in our earlier work (Wu et al., 2024). The experimental steps used were as follows: (I) activation and equilibration of the C18 SPE column; (II) slow adsorption of polyphenolic compounds; (III) elution to remove polar impurities; (IV) gradient elution and collection of target polyphenols. The obtained HH, HS, and HSC extracts rich in polyphenols were then lyophilized to form powders with a tan color. All operations related to polyphenols in the following experiments were carried out in the dark to prevent oxidation. In addition, the complete quantitative characterization data of the polyphenol extract adopted in this study, including high-performance liquid chromatography-tandem mass spectrometry (HPLC-MS/MS) chromatogram, total phenolic content, concentrations of major monomeric components and extract purity, are detailed in our previously published work (Wu et al., 2024). To eliminate the interference derived from inter-batch sample variation, all experiments in this study were performed with the same batch of lyophilized polyphenol powder.

### 2.4 Preparation of protein-phenolic conjugates

#### 2.4.1 Physical mixing

Physical mixtures were prepared by blending WP solution (1.0%, *w*/*v*) with HH, HS, or HSC solution (0.15%, *w*/*v*) using a magnetic stirrer (B13-3Pro, Shanghai Sile Instrument Co., Ltd., Shanghai, China) at room temperature (25 °C) and 500 rpm for 12 h. Subsequently, the mixed solutions were dialyzed (8 kDa cutoff) using DI water with incubation at 4 °C for a 48-h period to remove non-bound polyphenols and low-molecular-weight species. The resulting physical mixtures were designated as WP-HH-PM, WP-HS-PM and WP-HSC-PM, respectively.

#### 2.4.2 Heat treatment

Heat-treated protein-polyphenol conjugates were prepared based on the method described by Yan et al. (2022). Firstly, a WP solution (1.0%, *w*/*v*) was heated in a constant-temperature water bath (Grant JB Nova, Guangzhou Hongke Electronic Technology Co., Ltd., Guangzhou, China) at 80 °C for 15 min and then transferred rapidly to an ice-water bath to cool down to 25 °C. Subsequently, a HH, HS, or HSC solution (0.15%, *w*/*v*) was added, and the mixture was stirred on a magnetic stirrer at 500 rpm for 12 h. Then, the mixture was dialyzed (MWCO: 8 kDa) as described in Section 2.4.1, and the obtained samples were labeled as WP-HH-HT, WP-HS-HT and WP-HSC-HT respectively.

#### 2.4.3 Alkali treatment

Alkaline-treated protein-polyphenol conjugates were prepared following a method reported previously (Li et al., 2023). Firstly, HH, HS, or HSC solution (0.15%, *w*/*v*) was mixed with WP solution (1.0%, *w*/*v*) in phosphate buffer solution (PBS, 0.01 mol·L^−1^, pH 7.4). The pH of the mixed system was then tuned to 9.0 by adding 0.1 M NaOH dropwise. Subsequently, the samples were continuously stirred at 25 °C and 500 rpm for 24 h using a magnetic stirrer. The mixed solutions were then dialyzed (8 kDa) as described in Section 2.4.1. The resulting alkaline-treated conjugates were designated as WP-HH-AT, WP-HS-AT, and WP-HSC-AT, respectively.

#### 2.4.4 Free radical grafting

Free radical-induced covalent grafting was utilized to synthesize protein-polyphenol conjugates, adapted from the method established by Li et al. (2023). In brief, WP solution (1.0%, *w*/*v*) was mixed with 5 mmol·L^−1^ hydrogen peroxide (1%, *v*/*v*) and ascorbic acid (0.25%, *w*/*v*), then stirred magnetically at a temperature of 25 °C and 500 rpm for 2 h. Subsequently, HH, HS, or HSC solution (0.15%, *w*/*v*) was added, and the mixture was ultrasonicated using an ultrasonic cell disruptor (HN96-II, Shanghai Hanno Instrument Co., Ltd., Shanghai, China) while being maintained in the ice bath to prevent overheating. The mixture was then stirred under the same conditions for 24 h, followed by dialysis (8 kDa) as outlined in Section 2.3.1. The synthesized conjugates were coded as WP-HH, WP-HS, and WP-HSC-UFRGX, with the X parameter (1, 2, 3, 4) indicative of four ultrasonic processing conditions respectively: 200 W/10 min, 200 W/20 min, 400 W/10 min, and 400 W/20 min. The conjugates without ultrasonic treatment were named WP-HH/HS/HSC-FRG.

### 2.5 Characterization of the physical state and chemical structures of protein-polyphenol conjugates and complexes

#### 2.5.1 X-ray diffraction analysis

XRD analysis of the protein-polyphenol conjugates and complexes was conducted using an X-ray diffractometer (SAXSpoint 700, Anton Paar GmbH, Graz, Austria). Before the tests were conducted, the powdered samples were equilibrated in a vacuum desiccator at room temperature (25 °C) for 24 h. The testing conditions were set as follows: Cu-Kα radiation, 40 kV working voltage, 40 mA operating current, 2θ ranging from 5° to 60°, and a scan speed of 5°/min.

#### 2.5.2 Fourier transform infrared (FTIR) spectroscopy analysis

FTIR spectra of the protein-polyphenol conjugates were obtained using the KBr pellet method, according to the procedure reported by Yin et al. (2025). Briefly, 1 mg of the sample was blended with 100 mg of KBr and ground into a homogeneous powder using an agate mortar. Thereafter, the FTIR spectra were obtained using a PerkinElmer Frontier FTIR spectrophotometer (Waltham, MA, USA), with a scanning wavenumber range of 500–4000 cm^−1^ and a resolving power of 4 cm^−1^.

### 2.6 Characterization of microstructure for protein-polyphenol conjugates

#### 2.6.1 Scanning electron microscopy (SEM) analysis

The surface morphology of the lyophilized protein-polyphenol conjugate powder samples was characterized using a scanning electron microscope (SU9600, Hitachi Limited, Tokyo, Japan). Before performing imaging analysis, the samples were affixed onto silicon substrates and sputter-coated with a thin gold layer to ensure good electrical conductivity. Observations were then conducted under the following operational parameters: 15 kV acceleration voltage, 10–15 mm working distance, and 1000× magnification.

#### 2.6.2 Atomic force microscopy (AFM) analysis

The aggregation behavior of the protein-polyphenol conjugates and complexes was investigated using an atomic force microscope (Dimension Edge, Bruker Dimension Icon, Billerica, Massachusetts, USA). Initially, 5 μL of sample solution was dropped onto a freshly cleaved mica substrate and then air-dried under natural conditions. Imaging was performed using tapping mode at a resolution of 256 × 256 pixels. Finally, the instrument software (NanoScope Analysis V3.00, Bruker Corporation) was used for height quantification and topography analysis.

### 2.7 Raman spectroscopy analysis

Raman spectra of the protein-polyphenol conjugates and complexes were collected over the wavenumber range from 400 to 2200 cm^−1^ according to the method described by Zhai et al. (2025). The measurement parameters of the Raman spectrometer (HR8000, Hitachi Limited, Tokyo, Japan) were as follows: laser power 100 mW, acquisition time 60 s, excitation wavelength 785 nm, and spectral resolution 2 cm^−1^. All spectra were normalized relative to the phenylalanine (Phe) band observed at 1003 cm^−1^. The deconvolution of amide-I band (1600–1700 cm^−1^) for secondary-structure quantification was performed using Origin 2022 software. A baseline correction was conducted for background subtraction, followed by smoothing for noise reduction. Curve fitting adopted mixed Gaussian-Lorentzian peak-shape functions and four component peaks were assigned: α-helix (1645–1660 cm^−1^), β-sheet (1670–1680 cm^−1^), β-turn (1640–1644 cm^−1^/1680–1690 cm^−1^), random coil (1660–1670 cm^−1^). Peak positions were constrained within the corresponding wavenumber windows, while peak width and amplitude were freely optimized. Goodness-of-fit was assessed by adjusted coefficient of determination (R^2^) and residual sum of squares (RSS). All amide-I fittings reached R^2^ > 0.99. For disulfide-bond conformation analysis in the S—S stretching region (500–550 cm^−1^), identical baseline correction and smoothing routines were applied. The sub-peaks with mixed Gaussian-Lorentzian functions were fitted, corresponding to three disulfide-bond conformations: g-g-g (500–515 cm^−1^), g-g-t (515–525 cm^−1^), and t-g-t (525–550 cm^−1^). Peak positions were confined to the respective wavenumber windows, and peak width and amplitude were freely optimized. The relative percentage of each disulfide-bond conformation was calculated from integrated sub-peak area. All disulfide-bond fittings also achieved R^2^ > 0.99. Three independent replicate Raman spectra were acquired for every sample to evaluate fitting robustness.

### 2.8 Grafting degree determination

Quantitative determination of grafting degree was performed for the protein-polyphenol conjugates using a modified Folin-Ciocalteu method. The sample powder was first dispersed in DI water at a concentration of 2 mg·mL^−1^ and then stirred at 25 °C for 30 min. Thereafter, 0.5 mL aliquot of the suspension was combined with 0.5 mL Folin-Ciocalteu reagent, and the mixture was placed in darkness for continuous stirring over 1 h. Termination of the reaction was achieved by introducing 2 mL of 7.5% (*w*/*v*) sodium carbonate solution. After intensive shaking, the mixture was permitted to stand statically for 2 h. Subsequently, the absorbance of the solution was measured at 650 nm using a UV–Vis spectrophotometer. A standard curve was prepared by measuring the absorbance of gallic acid over a range of concentrations. The formula provided below was utilized to calculate the grafting rate:

(1) $$\mathrm{Grafting rate}\;\left(\%\right)=\frac{C}{c_{0}}\times 100\%$$

Here, *C* represents the polyphenol content grafted onto walnut protein (determined after dialysis) and *C*_*0*_ represents the initially added polyphenol content. The grafting extent was expressed as milligrams of polyphenol equivalents bound per gram of conjugate.

### 2.9 Turbidity analysis

The turbidity of protein-polyphenol conjugates and complexes in aqueous solutions was determined using the method reported by Wang et al. (2024), with minor modifications. In briefly, each conjugate solution was diluted to 3 mg/mL using DI water. Absorbance at 600 nm was determined with a UV–Vis spectrophotometer (UV765PC, Anhui Wanyi Technology Co., Ltd., Hefei, China), and DI water was used as the blank sample for calibration. The turbidity was then calculated with the formula listed below:

(2) $$\mathrm{Turbidity}\;\left(\%\right)=\frac{2.302\times A_{600}\times \mathrm{DF}}{L}\times 100\%$$

Here, *A*_*600*_ is the “absorbance” measured at 600 nm, *DF* is the dilution factor, and *L* is the optical path length of the cuvette (cm).

### 2.10 Surface wetting properties of protein-polyphenol conjugates

#### 2.10.1 Surface hydrophobicity analysis

The 8-anilino-1-naphthalenesulfonic acid (ANS) fluorescence probe assay was applied to quantify the surface hydrophobicity (H₀) of different samples, with minor revisions made to the method described by Miao et al. (2026). Specifically, 40 μL of 4 mM ANS solution was added to 4 mL of protein sample solution. After vortexing for 10 s, the mixture was left to equilibrate in the dark for 3 min. The fluorescence intensity was then measured with a fluorescence spectrophotometer (F-7100, Hitachi Limited, Tokyo, Japan) at an excitation wavelength of 390 nm and an emission wavelength of 470 nm. The H_0_ value was then calculated from the initial slope of the fluorescence intensity *versus* protein concentration curve obtained by linear regression analysis.

#### 2.10.2 Contact angle analysis

The contact angle (θ) of powdered protein-polyphenol conjugates or complexes against water was measured using an optical contact-angle analyzer (DM-701, Kyowa Interface Science Co. Ltd., Niiza-shi Saitama, Japan) (Li, Wu, et al., 2025). The powder samples were pressed into uniform wafers (5.5 mm in diameter and 1.5 mm in thickness). Subsequently, each wafer was brought into contact with a 5-μL static water droplet and the water-droplet profile was recorded and digitized using a high-speed CCD camera.

### 2.11 Statistical analysis

All experiments were conducted with a minimum of three independent replicates, and the results are expressed as mean ± standard deviation. Statistical significance was assessed by one-way analysis of variance (ANOVA) followed by Duncan's multiple range test (*P* < 0.05) using IBM SPSS Statistics 27 (IBM Corporation, Chicago, USA). Pearson correlation analysis was performed using the OmicStudio online tool (https://www.omicstudio.cn/tool). For Pearson correlation analysis, individual biological replicate data (total *n* = 48; 3 replicates for each of the 16 treatment groups) were used instead of averaged group-level treatment means. The 95% confidence intervals (CI) of correlation coefficients were calculated, and the Bonferroni correction was applied for multiple-testing adjustment to control family-wise error rate. Adjusted *P* < 0.05 was defined as statistically significant. Data visualization was performed with Origin 2022.

## 3 Results and discussions

### 3.1 Interaction mechanisms of walnut protein-hickory polyphenol complexes/conjugates

The processed mechanisms involved in conjugate and complex formation between walnut proteins and hickory polyphenols as mediated by the four treatment techniques are shown schematically in Figs. 1E-H. For physical mixing, the proteins should maintain their original native globular conformation and only engage in weak non-covalent interactions with the polyphenols (such as hydrogen bonding and hydrophobic interactions). For the heat treatment, the holding temperature used (80 °C) would be expected to promote moderate unfolding of the globular proteins, exposing more polar and non-polar sites, which could promote increased binding of polyphenols through non-covalent interactions. For the alkaline treatment (pH 9), the polyphenols would be expected to become oxidized to *ortho*-quinone intermediates, which can then form covalent conjugates with proteins through Michael addition and Schiff base reactions (Pi et al., 2023; Yan et al., 2023). For free-radical grafting, an ascorbic acid‑hydrogen peroxide redox system was used to generate hydroxyl radicals, which may have promoted appreciable protein unfolding. Moreover, covalent grafting of polyphenols should occur through radical coupling. Thus, both the conformation and surface chemistry of the protein molecules should be altered appreciably using this approach (Yan et al., 2023). As each of these processing methods leads to different changes in protein conformation, surface chemistry, and interactions, we hypothesized that they would lead to complexes and conjugates with different functional attributes. Nevertheless, it should be noted that no polyphenol-free protein blank control matched to each treatment condition was established in this study. Accordingly, all observed changes in conformation and physicochemical properties represent the superimposed effects of processing-triggered protein modification and protein-polyphenol intermolecular interactions.

### 3.2 Crystallographic properties and chemical structure of protein-polyphenol complexes/conjugates

In general, X-ray diffraction (XRD) can be used for identifying the structural characteristics (crystalline/amorphous) of materials and providing insights into their molecular packing. As shown in Fig. 2A, two relatively broad diffraction peaks were observed for the walnut protein powders at 9.85° and 19.46°, which suggests that they had an amorphous structure (Li et al., 2025). Furthermore, all the powdered protein-polyphenol complex and conjugate samples exhibited broad and diffused peaks rather than distinct sharp peaks (Fig. 2A-C), which indicated that they also had an amorphous structure. However, a diffraction peak emerged around 30°, which may be due to the formation of some locally structured areas, *e.g.,* hydrogen-bonded proteins and polyphenols. Moreover, the rigid bicyclic lactone backbone of ellagic acid and the flexible C-ring of rutin are known to contribute to a certain degree of structural order (Dávalos et al., 2019; Sanver et al., 2020). Notably, under radical-induced grafting conditions, the peak near 30° in the WP-HH system vanished, which may be due to the oxidation of the hydroxyl groups in the rutin molecules by free radicals and the breakage of unsaturated bonds (Afanas' ev et al., 1989). Furthermore, the diffraction peak intensity of the WP-HS-UFRG4 sample decreased significantly, indicating a remarkable reduction in the crystallinity of the sample. This phenomenon can be attributed to the disruption of the intrinsic structure (bicyclic lactone) and intermolecular hydrogen bonding network of ellagic acid by ultrasound, which generates high-frequency and high-intensity impact, shear, and thermal effects (Man et al., 2025). These results indicate that ultrasonic processing can alter the interactions between hickory polyphenols and walnut proteins, which may lead to changes in the structure of the powders formed.

**Fig. 2:**
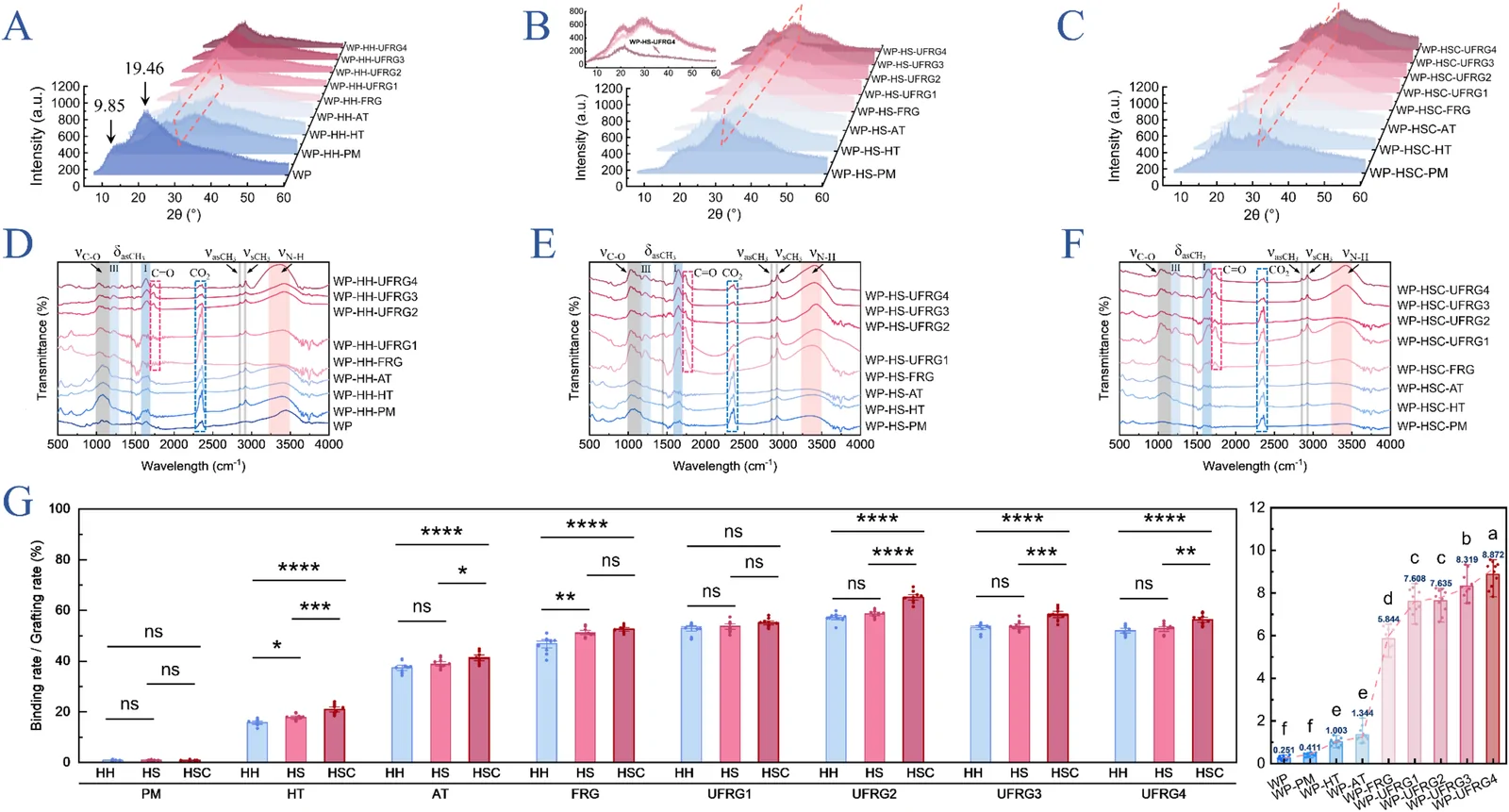
The crystal, chemical structural characteristics and polyphenol binding/grafting rate of the different WP-HH, HS and HSC complexes. (A) X-ray diffraction; (B) FTIR spectroscopy; (C) polyphenol binding/grafting rate and apparent Folin-Ciocalteu background values of polyphenol-free walnut protein subjected to different treatments. WP-PM: untreated native walnut protein; WP-HT: heat-treated protein only; WP-AT: alkali-treated protein only; WP-FRG: free-radical-treated protein only; WP-UFRG1–4: ultrasound-assisted free-radical-treated protein only with varied ultrasonic parameters.

Fourier transform infrared (FTIR) spectroscopy can be utilized to elucidate the functional groups and conformation of proteins, as well as to provide insights into the potential molecular interactions between hickory polyphenols and walnut proteins. As series of characteristic peaks were observed in the FTIR spectra (Fig. 2D of the pure walnut proteins, which can be attributed to different functional groups: 3444.29 cm^−1^ (amide A band), due to N—H stretching vibration; 1656.58 cm^−1^ (amide I band), due to C=O and COO^−^ stretching vibrations; 1257.38 cm^−1^ (amide III band), due to N—H bending vibrations and C—N stretching vibrations; and, 1064.53 cm^−1^ due to C—O stretching vibrations (Wang, Chen, et al., 2023; Yuan et al., 2024; Wei et al., 2025). In addition, all composite samples exhibited a weak absorption peak at 1457.94 cm^−1^ (Fig. 2D-F), which was attributed to the in-plane C—H bending vibration of the aromatic rings in the polyphenols (Huang et al., 2025). The absorption signals observed at 2854.43 and 2925.68 cm^−1^ were attributed to superimposed C—H stretching vibrations of aliphatic side chains in the proteins and polyphenols (Lian et al., 2025). A prominent peak was observed at 1739 cm^−1^ in all the covalently grafted samples, which was attributed to the C=O stretching vibration of the ester group (-O-CO-) (Wei et al., 2024). It should be noted that a single FTIR band near 1739 cm^−1^ cannot serve as definitive proof of ester-bond formation, as free carboxylic acids, native esters and polyphenol oxidation products can also absorb in this spectral region. Nevertheless, the prominent 1739 cm^−1^ peak was exclusively observed in free-radical-grafted samples (FRG/UFRG), while this feature was absent for physical-mixing, heat-treatment and alkali-treatment groups. According to our previously published FTIR characterization of the starting SPE-US-purified hickory polyphenol extracts, the raw polyphenol materials themselves show no obvious absorption at 1739 cm^−1^, ruling out intrinsic ester moieties from polyphenol feedstock (Wu et al., 2024). Furthermore, alkali-treated samples with abundant quinone-oxidized polyphenols also lacked this peak, which makes quinone-type oxidation products an unlikely source. In addition, a red-shift was observed in the C—O stretching vibration band of all the free-radical grafting systems, which was attributed to the interaction between the π-electron cloud of the aromatic ring and the p-π conjugate system of the ester group. Notably, compared with WP, the peak intensity of the amide I band in all the complexes was enhanced, which originated from the alteration in the electron-cloud distribution of the peptide-bond carbonyl group caused by the binding of proteins and polyphenols, directly verifying the formation of non-covalent or covalent interactions between them. According to previous research, there are differences in the main phenolic monomers in different extracts (Wu et al., 2024). Specifically, HH polyphenols (mainly rutin without carboxyl groups) interacted with proteins through weak hydrogen bonds, while HS and HSC polyphenols, which are rich in carboxyl groups, bind to proteins through strong hydrogen bonds as well as covalent ester or amide bonds. These intermolecular interactions were able to induce the unfolding of protein polypeptide chains and the rearrangement of the aggregation state. As a result, the local chemical micro-environment and bonding characteristics of the amide bonds on the protein surface were altered.

In conclusion, walnut proteins and hickory polyphenols formed powders with amorphous structures. The proteins and polyphenols within these powders were held together by non-covalent interactions (such as hydrogen bonding) or covalent interactions (primarily ester bonds). The FTIR results also show that the binding between walnut protein and different hickory polyphenols changed the peptide bond microenvironment and induced polypeptide chain unfolding as well as aggregation rearrangement, and the interaction patterns differed with polyphenol components.

### 3.3 Analysis of the polyphenol grafting degree

The grafting degree provides an indicator of the extent of conjugation of proteins and polyphenols. WP-PM derived solely from processed modified walnut protein exhibited a low apparent background value (0.251) (Fig. 2G). Heat treatment (WP-HT, 0.411) and alkali treatment (WP-AT, 1.003) only slightly elevated the background absorbance. In contrast, free-radical oxidation could significantly enhance the Folin-Ciocalteu signal of walnut protein even in the absence of polyphenols (WP-FRG, 5.844). For the UFRG series, the background signal progressively increased with rising ultrasonic power and prolonged ultrasonic time: WP-UFRG1 (7.608), WP-UFRG2 (7.635), WP-UFRG3 (8.319), WP-UFRG4 (8.872). These results confirm that oxidative processing (free-radical treatment combined with ultrasound) can oxidize intrinsic tyrosine and tryptophan residues within walnut protein, generating substantial color-forming reactions independent of polyphenols. The alkaline treatment group exhibited significantly higher grafting efficiency than the heat treatment group, which can be attributed to the efficient amidation reaction (-CO-NH-) that occurs between protein amino groups (-NH₂) and polyphenol carboxyl groups (-COOH) under strongly alkaline conditions. Notably, grafting was still observed for the HH group (rutin) even though it did not have carboxyl groups, which may have been due to the following mechanism: (I) multiple phenolic hydroxyl groups (-OH) in rutin were ionized into phenoxide anions (—O) under alkaline conditions, and these anions then formed ion-dipole interactions with the amino groups (-NH_3_^+^) of the protein; (II) the alkaline treatment led to partial unfolding of the protein due to an increase in intra-molecular electrostatic repulsion (Li et al., 2023). The glycosyl groups (polyhydroxyl structures) and flavonoid aglycones of rutin could be extensively bound to these exposed sites through hydrogen bonds (Sanver et al., 2020). In the free radical grafting group, as the ultrasonic power and duration increased, the grafting efficiency first increased and then decreased. The highest grafting efficiency was achieved under the conditions of 200 W and 20 min. This indicated that within a certain range, the mechanical effects of ultrasound (cavitation, shearing) could enhance free-radical reactions, promote molecular contact between proteins and polyphenols, and thus improve the grafting efficiency. Meanwhile, appropriate ultrasonic treatment could unravel the rigid lactone structure in hickory polyphenols, disrupt their intramolecular hydrogen bonds, expose more phenolic hydroxyl and carboxyl groups, and further strengthen the binding between proteins and polyphenols. However, excessive ultrasonic power and treatment time might lead to the oxidative degradation of the aromatic rings of polyphenols, which could cause the loss of some reaction activity, the breakage of covalent bonds, and a reduction in the number of active binding sites, ultimately weakening the interaction between proteins and polyphenols. Therefore, hickory polyphenols can be loaded onto walnut proteins efficiently *via* the UFRG2 treatment (200 W/20 min), which can enhance the bioavailability of polyphenols, mask their bitterness, and thereby improve the sensory acceptance and application potential of this complex in actual food matrices.

### 3.4 Microstructure of protein-polyphenol complexes/conjugates

The surface morphologies of walnut proteins and protein-polyphenol conjugates were observed using scanning electron microscopy (SEM) and atomic force microscopy (AFM), as shown in Fig. 3. The SEM images of the walnut protein powders showed that they contained smooth or slightly wrinkled spheroidal particles. The SEM images of the powdered physically mixed and heat-treated groups showed that they contained numerous flaky or irregular shaped structures. After the alkaline treatment, the SEM images showed that the particles in the powdered conjugate samples had a dense membrane-like structure with smooth surfaces. After the free-radical-grafting reaction, the samples had a flake-like structure. After the ultrasonic-FRG treatment, all the samples contained relatively small particles, rather than large irregular aggregation. This effect may have been because the high-intensity pressure waves were able to break up the larger aggregates (Yan et al., 2023).

**Fig. 3:**
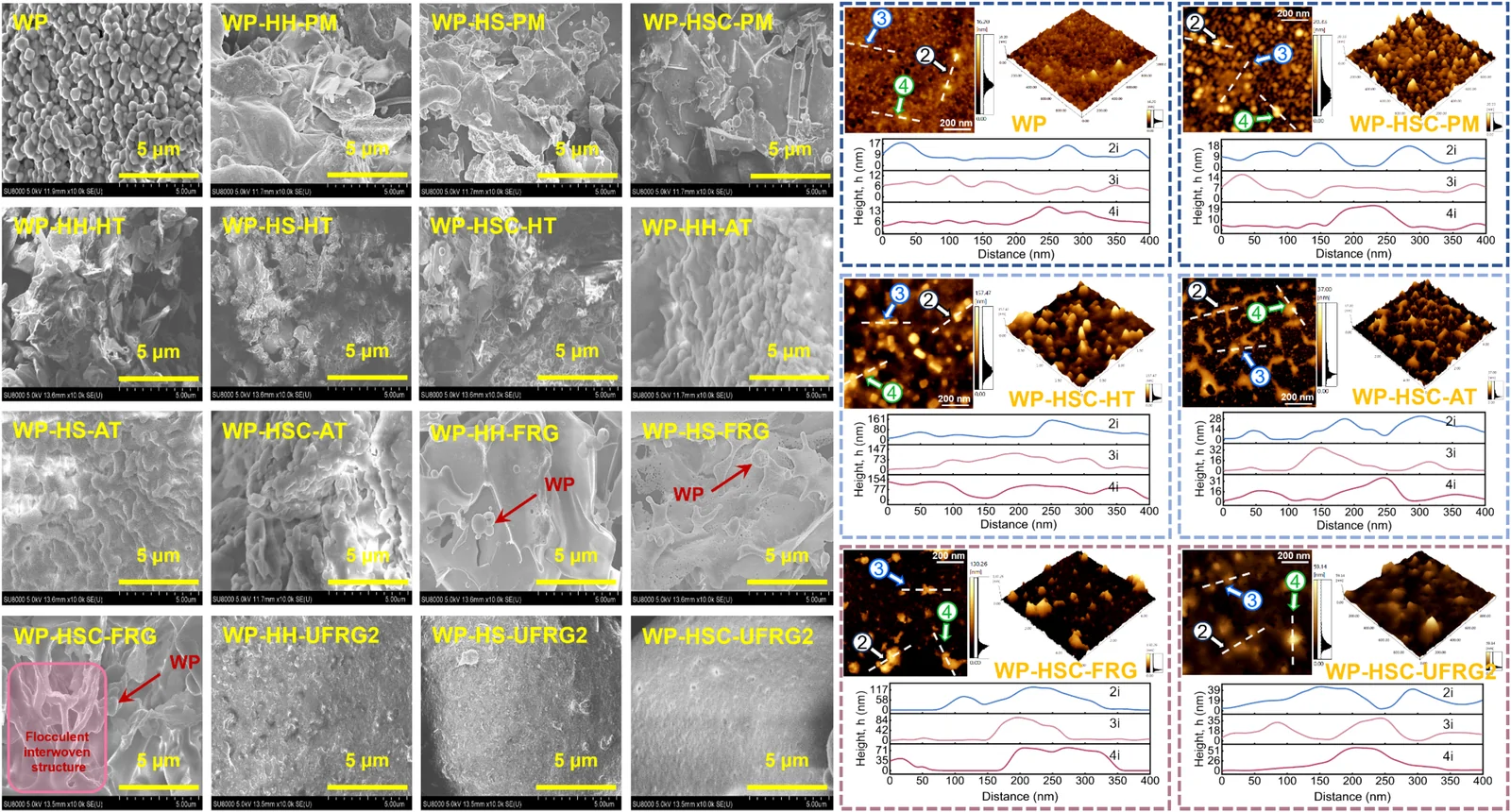
The scanning electron microscope (SEM) and atomic force microscopy (AFM) images of the different WP-HH, HS and HSC complexes.

Atomic force microscopy (AFM) was used to characterize the surface morphology of the different samples at the nanoscale after they were deposited on flat solid surfaces. The pure walnut protein sample had a relatively uniform and closely distributed particulate aggregation state, with a maximum height of around 16.2 nm. For the physically mixed group, the particle size increased slightly compared to the pure proteins, and some larger aggregates were observed, with the maximum height increasing to around 20.3 nm. For the heat-treated group, there was evidence of strong aggregation, as the maximum height increased to around 157.5 nm, which may have been because heating promoted thermal unfolding and association of the proteins. For the alkali treatment, there was also an increase in the degree of aggregation compared to the original proteins, with the maximum height being around 47 nm, which may be due to some pH-induced unfolding and association of the proteins. Notably, the WP-HSC-FRG conjugates had numerous heterogeneous patchy protrusions in their images (maximum height: 130.3 nm), suggesting that it also promoted aggregation. However, ultrasonication appeared to reduce the degree of aggregation (maximum height: 59.1 nm), which can be attributed to dissociation of the aggregates by the high-intensity pressure waves. However, this structural characteristic highlights the unique advantage of UFRG compared with HT, AT, and FRG. The smaller and more uniform aggregates formed under UFRG are more conducive to the dispersion of protein-polyphenol complexes in aqueous food systems, avoiding precipitation or phase separation caused by large aggregates, which is critical for their application in beverages, emulsions and other liquid foods.

### 3.5 Raman spectroscopy of protein-polyphenol complexes/conjugates

Raman spectroscopy was used to investigate the molecular structure and composition of the different samples based on frequency shifts in the inelastic scattered radiation. Special attention was paid to the fingerprint region of 500–1800 cm^−1^, where protein amide I-III vibrations and polyphenol aromatic ring patterns are known to display strong Raman activity, enabling monitoring of any conformational changes and interactions in the conjugates.

As shown in Fig. 4A-D, the pure walnut protein exhibited several characteristic Raman bands: (I) amide I (1600–1700 cm^−1^, C=O stretching) and amide III (1230–1300 cm^−1^, C—N stretching coupled with N—H bending) regions; (II) hydrophobic aliphatic amino acid side chains manifested through C—H bending vibrations at 1380 cm^−1^ and 1450 cm^−1^; (III) tyrosine (850 cm^−1^), tryptophan (760 cm^−1^), and disulfide conformers (500–550 cm^−1^). These spectral features provide insights into the secondary structure and side chain microenvironmental alterations:

**Fig. 4:**
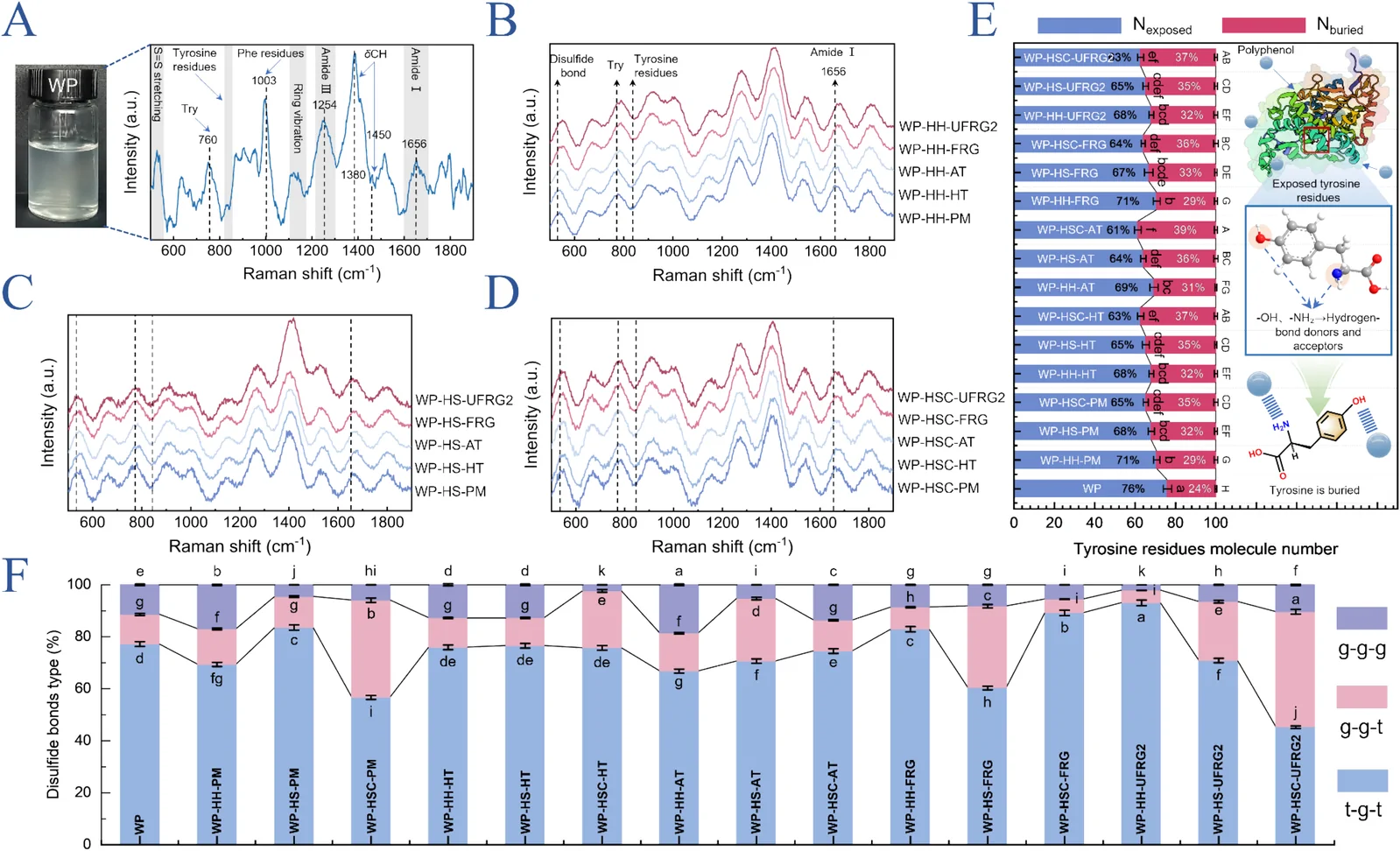
Protein conformational changes of the different WP-HH, HS and HSC complexes. (A) Raman spectrum; (B) ratio of exposed and buried tyrosine residues; (C) different disulfide-bond modes.

#### 3.5.1 Secondary structure of conjugates

Based on deconvolution and curve-fitting analysis of the amide I band (1600–1700 cm^−1^) region, the relative contents of α-helix, β-sheet, β-turn, and random coil structures were quantified (Table 1, Fig. S1). The walnut protein alone was dominated by ordered conformations such as α-helix (31.48%) and β-sheet (22.89%) structures. For the physically mixed groups, the α-helix content of all the systems (HH, HS, and HSC) was higher than that of WP, while the β-sheet content was slightly less. This effect indicated that polyphenols stabilized the α-helix structure of the proteins, possibly through weak non-covalent interactions (such as hydrogen bonding and hydrophobic associations) without causing significant protein unfolding. For the heat-treated groups, the α-helix content was lower than pure WP, while the random coil content was higher. This effect could lead to increased exposure of buried hydrophobic domains and hydrogen-bonding sites in the protein molecules, which enhanced polyphenol-binding and non-specific protein-protein aggregation. Consequently, it would account for the increased aggregation of the WP-HSC-HT samples seen in the AFM images.

**Table 1:** Secondary structure contents, side-chain group band intensities of the different WP-HH, HS and HSC complexes.

Samples	Secondary structure content (%)				Band intensity of side chain groups		
	α-Helix	β-Sheet	β-Turn	Random coil	Trp (I_760_/I_1003_)	Tyr (I_850_/I_830_)	CH (I_1450_/I_1003_)
WP	31.48 ± 0.88^bc^	22.89 ± 0.62^c^	22.57 ± 0.70^efg^	23.06 ± 0.84^g^	0.85 ± 0.03^e^	1.07 ± 0.04^a^	0.84 ± 0.03^c^
WP-HH-PM	37.97 ± 0.72^a^	19.76 ± 0.67^de^	23.68 ± 0.52^def^	18.56 ± 0.41^h^	0.97 ± 0.02^cd^	1.03 ± 0.03^ab^	1.03 ± 0.03^b^
WP-HH-HT	26.32 ± 1.08^ef^	22.17 ± 0.42^c^	26.76 ± 1.15^c^	24.75 ± 0.77^fg^	0.99 ± 0.04^cd^	1.01 ± 0.02^ab^	1.02 ± 0.02^b^
WP-HH-AT	33.11 ± 0.76^b^	10.75 ± 0.44^g^	32.88 ± 0.59^a^	23.25 ± 0.53^g^	0.96 ± 0.02^d^	1.02 ± 0.04^ab^	1.05 ± 0.04^b^
WP-HH-FRG	29.32 ± 1.06^cd^	22.06 ± 0.51^c^	20.63 ± 0.76^gh^	27.99 ± 1.12^ef^	0.97 ± 0.03^cd^	1.03 ± 0.02^ab^	1.05 ± 0.02^b^
WP-HH-UFRG2	27.87 ± 0.59^de^	3.24 ± 0.15^i^	19.86 ± 0.52^h^	38.27 ± 0.95^c^	1.06 ± 0.03^abc^	1.01 ± 0.03^ab^	1.06 ± 0.05^b^
WP-HS-PM	38.01 ± 1.67^a^	21.32 ± 0.66^cd^	23.57 ± 0.94^de^f	17.10 ± 0.68^h^	0.99 ± 0.04^cd^	1.01 ± 0.01^ab^	1.00 ± 0.02^b^
WP-HS-HT	23.22 ± 0.39^gh^	22.34 ± 0.56^c^	24.13 ± 0.48^de^	30.13 ± 0.62^e^	1.01 ± 0.02^abcd^	0.99 ± 0.02^ab^	1.05 ± 0.03^b^
WP-HS-AT	27.62 ± 0.88^de^	29.36 ± 0.53^a^	9.07 ± 0.41^k^	33.96 ± 1.34^d^	1.00 ± 0.04^bcd^	0.98 ± 0.03^b^	1.04 ± 0.01^b^
WP-HS-FRG	23.86 ± 1.12^fg^	25.40 ± 0.97^b^	21.98 ± 0.73^fg^	28.76 ± 0.80^e^	1.01 ± 0.02^abcd^	1.00 ± 0.02^ab^	1.00 ± 0.03^b^
WP-HS-UFRG2	20.89 ± 0.52^h^	6.91 ± 0.30^h^	25.31 ± 0.61^cd^	46.88 ± 1.61^b^	1.09 ± 0.04^ab^	0.99 ± 0.04^ab^	1.20 ± 0.04^a^
WP-HSC-PM	33.41 ± 1.00^b^	15.10 ± 0.33^f^	31.89 ± 1.12^a^	19.60 ± 0.54^h^	1.01 ± 0.05^abcd^	0.99 ± 0.02^ab^	1.06 ± 0.02^b^
WP-HSC-HT	24.35 ± 0.44 fg	18.60 ± 0.65^e^	29.43 ± 0.47^b^	27.62 ± 0.97^ef^	0.99 ± 0.02^cd^	0.97 ± 0.03^b^	1.05 ± 0.03^b^
WP-HSC-AT	25.81 ± 1.08efg	28.91 ± 0.84^a^	14.70 ± 0.62^i^	30.57 ± 1.21^e^	1.02 ± 0.03^abcd^	0.96 ± 0.04^b^	1.02 ± 0.04^b^
WP-HSC-FRG	30.04 ± 0.66^cd^	16.23 ± 0.65^f^	14.35 ± 0.40^i^	39.38 ± 1.03^c^	1.01 ± 0.04^abcd^	0.98 ± 0.02^b^	1.00 ± 0.02^b^
WP-HSC-UFRG2	5.36 ± 0.19^i^	9.09 ± 0.19^g^	11.25 ± 0.44^j^	74.30 ± 2.46^a^	1.10 ± 0.02^a^	0.97 ± 0.03^b^	1.04 ± 0.03^b^

Mean values calculated from three independent replicate Raman spectral fittings; values within the same column followed by different superscript letters are significantly different (*P* < 0.05).

The amidation reaction between protein amino groups (-NH₂ → -NH₃^+^) and polyphenol carboxyl groups (-COOH→-COO^−^) can be activated by alkaline treatments. Moreover, alkaline treatments can promote protein unfolding, which increases the number of reactive sites available on the protein surfaces. These effects may account for the observed changes in secondary structure of the proteins for the alkaline treated samples. Notably, however, there were appreciable differences in the effects induced by the alkaline treatment for the different kinds of hickory polyphenols. For instance, there was an appreciable decrease in β-sheet and increase in β-turn for the HH samples, but the opposite for the HS and HSC samples (Table 1). HH (primarily composed of rutin) contains fewer carboxyl groups than HS and HSC, thereby leading to a greater reliance on phenolic hydroxyl groups to form hydrogen bonds with the protein. The absence of covalent bonds may cause the dissociation of the originally regular inter-sheet stacking arrangements, with partial transformation into β-turns, *i.e.,* local chain folding (Li et al., 2025).

For the free radical grafted groups without (FRG) or with (UFRG2) sonication, the content of random coils increased while that of the ordered conformations decreased, with more significant disordering observed in the ultrasound-assisted group. This effect indicated that free-radical-mediated ester-bond cross-linking disrupted the original hydrogen-bond network and regular secondary structure within the walnut proteins. Moreover, the ultrasonic treatment further promoted the disruption of the molecular conformation. These conformational rearrangements may further expose the reactive groups on the protein surfaces to the polyphenols, thereby enhancing the binding interactions.

#### 3.5.2 The C—H stretching vibration of conjugates

The CH ratio (I_1450_/I_1003_) in Raman spectroscopy serves as a key quantitative indicator for evaluating the microenvironment of aliphatic amino acid side chains and characterizing hydrophobic interactions in protein structures. As shown in Table 1, the CH ratios of all treatment groups were higher than WP (0.84), indicating that the interactions between hickory polyphenols and walnut proteins could promote the exposure of aliphatic amino-acid side chains from the internal hydrophobic cavities of the protein to the outside and enhance the hydrophobic interactions between side chains (Zhai et al., 2025). Notably, the CH ratio of the WP-HS-UFRG2 increased to 1.20, indicating a significant transformation of aliphatic amino-acid side chains into the “exposed state” (Kang et al., 2025). This change was associated with the unfolding of the walnut protein structure. After exposure, these side chains further enhanced the C—H vibrational activity through molecular association, possibly driven by hydrophobic interactions. Although the random coil content in the WP-HSC-UFRG2 was as high as 74.30%, the CH ratio (1.04) was still lower than in the WP-HS-UFRG2 (1.20) which could be because excessive structural disorder induced hydrophobic aggregation, causing the initially exposed side chains to be re-buried through a strong hydrophobic attraction effect.

#### 3.5.3 Changes in the microenvironment of aromatic amino acids

The normalized intensity ratio (I_760_/I_1003_) can be used to characterize the microenvironment of tryptophan residues and monitor changes in their hydrogen bond states. The tryptophan ratios of all the treatment groups (0.96–1.02) were higher than pure WP (0.85), indicating that tryptophan residues were exposed from the embedded hydrophobic environment to a more polar environment by the polyphenol-induced structural rearrangement (Wang et al., 2023). In the ultrasound-assisted radical grafting (UFRG2) system, ultrasonic cavitation and shear effects disrupted the intramolecular hydrogen-bond network inside hickory polyphenol molecules, which improved molecular flexibility and exposed more hydrophobic aromatic domains and active binding sites. Such non-covalent interactions within polyphenols strengthened hydrophobic association and interfacial binding between polyphenols and the internal hydrophobic cavities of walnut protein, which is consistent with the interaction characteristics of protein-polyphenol complexes. Importantly, the spectral observations from this study reflect microenvironmental alterations of protein-derived aromatic amino-acid side chains (tryptophan and tyrosine) induced by polypeptide conformational rearrangement, which are insufficient to demonstrate chemical cleavage occurring on the aromatic carbocyclic skeletons of protein or polyphenol molecules. Nevertheless, enhanced polyphenol-protein interactions further trigger polypeptide-chain unfolding of walnut protein, which leads to the disruption of ordered secondary-structure arrangements. It has been reported that a decrease in β-sheet content of proteins is negatively correlated with hydrophobic interactions (Feng et al., 2025). The tryptophan ratios of the WP-HH-UFRG2 (β-sheet: 3.24%), WP-HS-UFRG2 (6.91%) and WP-HSC-UFRG2 (9.09%) reached 1.06, 1.09 and 1.10 respectively, which suggested that disruption of the β-sheet structure was correlated to an increased exposure of tryptophan residues. Conversely, samples with higher β-sheet contents (WP-HS-AT: 29.36%, WP-HH-PM: 19.76%) exhibited moderate-to-low tryptophan intensity ratios (1.0 and 0.97) and high CH ratios (1.04 and 1.03). This inverse relationship may have been because β-sheet structures can stabilize the hydrophobic cavities in globular proteins, thereby restricting the contact of tryptophan residues with the surrounding aqueous solvent. Overall, these findings suggest that the UFRG2 treatment disrupted the β-sheet structures in the walnut proteins and increased the exposure of hydrophobic groups to water.

The intensity ratio of tyrosine bands (I_850_/I_830_, Fermi doublet) can be used to identify the hydrogen-bonding state of phenolic hydroxyl groups in tyrosine residues (Table 1). The I_850_/I_830_ ratio for walnut protein alone (1.07) was slightly higher than that of all the protein-polyphenol groups (0.96–1.03), which may have been because the number of phenolic hydroxyl groups in the tyrosine residues that acted as hydrogen-bond acceptors increased. To determine whether the tyrosine residues in the proteins were exposed or buried, the relative exposure level was quantified using the following relationship:

$$N_{\mathrm{buried}}+N_{\mathrm{exposed}}=1;0.5N_{\mathrm{buried}}+1.25N_{\mathrm{exposed}}=I_{850}/I_{830}$$

Here, *N*_buried_ and *N*_exposure_ represent the mole fractions of buried and exposed tyrosine residues, respectively. As shown in Fig. 4E, the *N*_exposed_ value for the pure walnut protein was the highest (76%), indicating that the tyrosine residues were most exposed in the absence of polyphenols. Meanwhile, the hydroxyl oxygen atoms on the ring can act as donors and acceptors of medium-strength hydrogen bonds (Feng et al., 2025; Kang et al., 2025). The fact that the *N*_exposed_ values of all the polyphenol-treated groups were lower than that of the pure walnut proteins suggests that the interactions between the polyphenols and proteins may have promoted the conversion of tyrosine residues from an exposed state to a buried state. Notably, there were differences in the number of exposed and buried tyrosine residues for the different treatments, which again suggests that they had different effects on protein conformation, surface chemistry, and aggregation state.

#### 3.5.4 Disulfide bonds and sulfhydryl groups in complexes/conjugates

Disulfide bonds (formed by two cysteine residues) play a crucial role in determining the stability of the tertiary structure of globular proteins. In general, disulfide bonds may adopt different isomeric conformations: *gauche-gauche-gauche* (g-g-g), *gauche-gauche-trans* (t-g-g) and *trans-gauche-trans* (t-g-t). The percentage distribution of disulfide bond conformations across all investigated samples is summarized in Fig. 4F. Pure walnut protein was mainly in the t-g-t conformation (77.2%), which corresponds to the disulfide-bond cross-linking inside the protein molecules. This structural motif could “lock” the folding of the polypeptide chain into a compact globular structure and was consistent with the relatively high proportion of ordered secondary structure of WP (Table 1 and Fig. S2). However, in the physically mixed group, the carboxyl groups (-COOH) in the HSC can form strong hydrogen bonds with the amino groups (-NH₂) and hydroxyl groups (-OH) on the surface of the protein molecules, disrupting the hydrophobic interactions inside the protein's hydrophobic cavity. This disruption potentially led to the breakage of the intramolecular t-g-t conformation disulfide bonds that maintained the globular structure and induced the formation of a large number of flexible g-g-g and g-g-t conformations.

For the heat treatment conditions, the t-g-t conformations of WP-HH and WP-HS reached 76.0% and 76.5% respectively, approaching the level of WP. This effect indicated that disulfide bonds tend to be reconstructed into the thermodynamically stable t-g-t conformation. In contrast, the WP-HSC-HT sample retained a considerable proportion of the g-g-t conformation (21.9%), suggesting that the WP-HSC polyphenol system still maintained partial conformational flexibility during thermal processing.

For the alkaline treated group, all samples maintained a relatively high t-g-t conformation (66.8–74.5%), which may be related to the restriction of the conformational freedom of intermolecular disulfide-bond cross-linking by covalent bonds. Notably, the ·OH free radicals in the free-radical grafting (FRG) can drive the reconstruction of thiol-disulfide bonds. Both the WP-HH-FRG and WP-HSC-FRG samples were dominated by the t-g-t conformation (82.9% and 89.2% respectively), but WP-HS-FRG still retained 31.5% of the flexible g-g-t conformation. Ultrasonication further amplified the regulatory effect, as manifested by significant differences among the different polyphenol systems: HH (t-g-t: 93.1%) > HS (70.9%) > HSC (45.2%). This result indicates that the type of polyphenol functional groups present effect the polyphenol-protein interactions (thiol/disulfide exchange reactions) during ultrasonication.

Based on this analysis, the WP-HSC-UFRG2 was regarded as the desirable system for disulfide-bond conformation regulation. The g-g-t flexible conformation (44.4%) and t-g-t rigid conformation (45.2%) were almost equal for this sample. This conformational balance may help to maintain the structural stability of the proteins in solution through the rigid t-g-t conformation but also enhance the interfacial activity of the proteins through the flexible g-g-t conformation.

### 3.6 Analysis of turbidity

Turbidity measurements provide an indication of the impact of protein-polyphenol interactions on the aggregation behavior of aqueous dispersions. Generally, an increase in turbidity reflects the formation of either larger or more numerous aggregates. As shown in Fig. 5A, the turbidity of all treatment groups with added polyphenols was higher than that of pure walnut protein (20.9%), which indicated that the interactions (non-covalent/covalent binding) between the polyphenols and proteins promoted the aggregation of the protein molecules, thereby increasing the degree of light scattering (Wang et al., 2024). The turbidity of the different samples increased in the following order: WP < PM < AT < HT < FRG < UFRG2. This difference can be attributed to differences in the impact of the different polyphenols and processing treatments on the conformation and surface chemistry of the walnut proteins, which then altered their tendency to aggregate with each other. In the free-radical grafting system, the thiol groups (-SH) of proteins were attacked by hydroxyl radicals (·OH), generating sulfur radicals (·S-). The formation of ester covalent bonds between the carboxyl groups (-COOH) of polyphenols and the hydroxyl groups (-OH) of proteins was promoted (as confirmed by the characteristic peak at 1739 cm^−1^ in FTIR) and disulfide-bond rearrangement was triggered. Subsequently, highly cross-linked particles were formed (block-like aggregates were observed by SEM), thus increasing the turbidity.

**Fig. 5:**
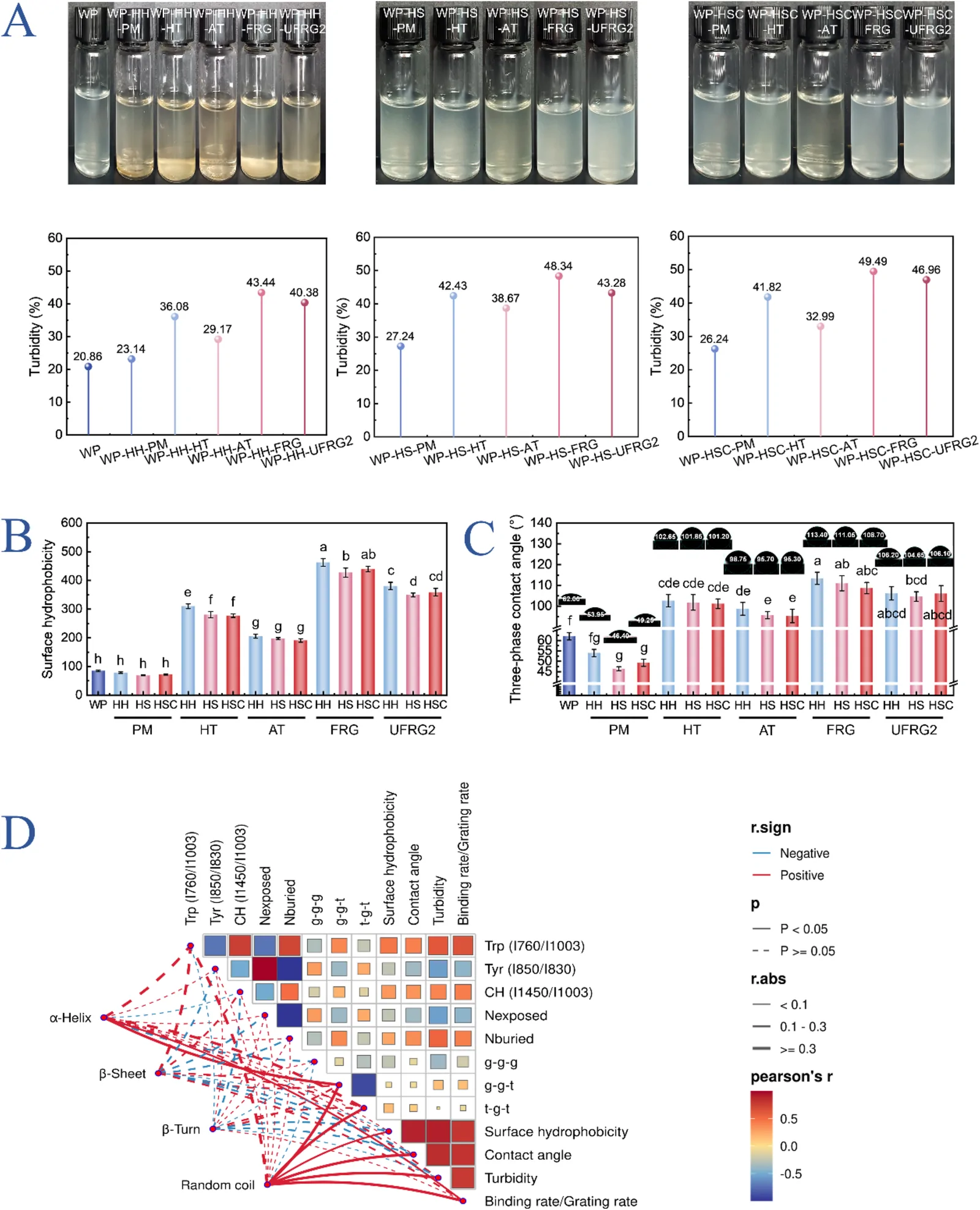
The (A) turbidity, (B) surface hydrophobicity, (C) contact angle, as well as (D) the correlation heatmap and correlation analysis of protein secondary structure, conformational parameters and physicochemical properties of the different WP-HH, HS and HSC complexes.

Notably, the ultrasound-assisted free-radical grafting (UFRG2) treatment had the highest turbidity, even though it contained smaller particles (as seen by AFM analysis). This effect may be because the turbidity of colloidal dispersions actually increases and then decreases as the particle size increases, with a maximum value occurring around a few hundred nanometers (McClements, 2002). Consequently, the UFRG2 samples may have had relatively small colloidal particles that scattered light more strongly than larger ones.

### 3.7 Analysis of surface hydrophobicity and interfacial wettability

The surface hydrophobicity (H_0_) provides a measure of the exposure of hydrophobic regions on protein surfaces, which can provide insights into the interactions between polyphenols and proteins. As shown in Fig. 5B, for the physically mixed groups, the addition of the different polyphenols (HH, HS, and HSC) caused a small (non-significant) decrease in surface hydrophobicity, which may have been because they bound to non-polar patches on the protein surfaces. The heat treatment and the alkali treatment bot promoted a substantial increase in surface hydrophobicity, with the effect being largest for the heat treatment. This effect can be attributed to the ability of high temperatures and strongly alkaline conditions to promote the unfolding of globular proteins, thereby leading to the exposure of hydrophobic groups at their surfaces. The higher surface hydrophobicity of the heat-treated group may have been because high temperatures promoted more extensive protein unfolding than high pH values (Li, Wu, et al., 2025; Wei et al., 2025).

The samples that had undergone the free radical grafting treatment, either without (FRG) or with (UFRG2) sonication, had considerably higher surface hydrophobicity values than the other samples. This effect may have been because the ·OH radicals disrupted the protein's structure, but also because they induced oxidation of the polyphenols to quinones. The hydrophobic properties of quinones synergized with the protein's hydrophobic residues, further enhancing the overall surface hydrophobicity. Notably, the ultrasonic treatment reduced the surface hydrophobicity of the proteins somewhat, which suggests that it may have promoted some reorganization of the protein molecules so that some of their exposed non-polar groups interacted with each other.

Changes in the contact angle of the different powdered samples were utilized to evaluate their hydrophilic-hydrophobic properties (Fig. 5C). The contact angles of the samples in the physically mixed group decreased compared with the walnut protein alone, which is indicative of a decrease in hydrophobicity, which is consistent with the surface hydrophobicity (H_0_) results discussed earlier. This effect can be attributed to the binding of polyphenols to the non-polar groups on the protein surfaces, thereby decreasing their surface hydrophobicity (Yan et al., 2024). As expected, a higher surface hydrophobicity (H_0_) corresponded to a higher contact angle for all the samples (Fig. 5B and C), so these two parameters followed similar trends: FRG > UFRG > HT > AT > PM ≈ WP. Notably, the contact angles of all treatment groups exhibited a consistent trend in terms of polyphenol type (*i.e.,* HH > HS > HSC), which can be attributed to differences in the number of polar and non-polar groups on the different polyphenols.

### 3.8 Correlation analysis

A correlation heat map was established among the protein secondary structure, the exposure of hydrophobic side-chain groups, the disulfide-bond conformation, surface hydrophobicity, contact angle and turbidity of the protein-polyphenol conjugates and complexes (Fig. 5D). The random coil content showed significant positive correlations (*P* < 0.05) with g-g-t, t-g-t disulfide-bond conformer fractions, surface hydrophobicity, contact angle, turbidity and polyphenol grafting efficiency. These correlations reflect concomitant co-variation among these variables under different modification treatments. Increased random-coil proportion, indicative of polypeptide-chain disorder, occurred alongside elevated surface hydrophobicity and higher grafting efficiency, likely due to exposure of formerly buried amino-acid side-chains. Nevertheless, these observed correlations do not establish causal relationships, because heat, alkali, free-radical and ultrasound treatments simultaneously alter multiple protein structural and physicochemical properties. Surface hydrophobicity was strongly positively correlated with contact angle and turbidity (*P* < 0.05). Higher turbidity co-varied with increased exposure of non-polar groups on protein surfaces, which may promote protein aggregation *via* hydrophobic attraction.

## 4 Conclusion

In this study, either conjugates or complexes were formed from walnut proteins and hickory polyphenols using a variety of processing methods, including simple mixing, heating, alkaline treatment, free-radical grafting, and ultrasonic-assisted free-radical grafting. The properties of the different protein-polysaccharide conjugates and complexes were then characterized using a range of complementary analytical methods. As expected, physical mixing caused only mild structural perturbations in the protein structure, whereas the other methods caused appreciable alterations in the conformation, surface chemistry, and aggregation state of the proteins. Therefore, based on these differences, this study elucidated the inherent relationships among polyphenol structures, processing conditions, and the interaction mechanisms between proteins and polyphenols, which provided the molecular theoretical basis for precisely regulating the structures of plant protein-polyphenol complexes and achieving the high-value utilization of hickory by-products. However, this study focused on the structural characteristics and interaction mechanisms of complexes/conjugates, without verifying the relevant functional properties in food applications, such as polyphenol solubility, specific bitterness-masking effect, processing stability, and bioaccessibility. Therefore, the performance and stability of such complexes/conjugates during the processing, storage, and digestion of actual food systems remain to be further explored.

## CRediT authorship contribution statement

**Shuaizheng Wang:** Writing – review & editing, Writing – original draft, Visualization, Validation, Supervision, Software, Methodology, Investigation, Formal analysis, Data curation, Conceptualization. **David Julian McClements:** Visualization, Validation, Supervision, Methodology, Conceptualization. **Hao Hu:** Visualization, Software, Data curation, Conceptualization. **Li Huang:** Validation, Supervision, Software. **Liang Cao:** Visualization, Validation, Supervision, Resources, Methodology, Investigation, Data curation, Conceptualization. **Changling Wu:** Writing – review & editing, Visualization, Validation, Supervision, Software, Resources, Project administration, Methodology, Investigation, Funding acquisition, Formal analysis, Data curation, Conceptualization.

## Declaration of competing interest

The authors declare that they have no known competing financial interests or personal relationships that could have appeared to influence the work reported in this paper.

## Data Availability

No data was used for the research described in the article.
