# Supplementary material for: Fabrication and characterization of walnut protein-polyphenol conjugates from hickory by-products: Structure and interaction mechanism analysis
Source: Food Chem X. 2026 Sep 21;39:104491. doi: 10.1016/j.fochx.2026.104491 (PMC13634361; doi:10.1016/j.fochx.2026.104491)
Supplement: Supplementary file 1 [file mmc1.docx]

# Supplementary material

Fig. S1. Peak-fitting diagram of protein secondary structure by Raman spectroscopy in the amide I region (1600-1700 cm^-1^)

Fig. S2. Peak-fitting diagram of protein disulfide bonds by Raman spectroscopy (500-550 cm^-1^)
